# An adult with hemorrhagic varicella co-infects with cytomegalovirus: a case report

**DOI:** 10.1186/s12879-024-09383-0

**Published:** 2024-07-11

**Authors:** Si-Man Shi, Zhan-Hong Li, Jie-Ru Xu, Xue-Xin Cai, Xiu-Xian Zhou, Rong-Chang Zheng, Ju Wen

**Affiliations:** 1 The Affiliated Guangdong Second Provincial General Hospital of Jinan University, Guangzhou, 510317 China; 2 Guangdong clinical college of dermatology, Anhui medical university, Guangzhou, 510000 China; 3 Guangdong Medical University, Zhanjiang, 524023 China

**Keywords:** Adults, Hemorrhagic varicella, Nephrotic syndrome, Cytomegalovirus, Infection, Case report

## Abstract

**Background:**

Hemorrhagic varicella (HV) is a particular form of chicken pox.,with high mortality in adults. This form of the disease is rare, to date, approximately 4 cases have been reported. Occasional cases of HV have been documented in adults with hematologic disorders or other diseases. While there is one reported case of simultaneous reactivation of cytomegalovirus in an adult with chickenpox, there is a lack of information regarding changes in liver function indicators for such patients. This is unfortunate, as CMV reactivation can further exacerbate liver failure and increase mortality. In this report, we present a case of hemorrhagic varicella reactivation with cytomegalovirus and provide some relevant discussions.

**Case presentation:**

We present the case of a 25-year-old male with HV, who had a history of nephrotic syndrome generally controlled with orally administered prednisone at a dosage of 50 mg per day for two months. The patient arrived at the emergency room with complaints of abdominal pain and the presence of hemorrhagic vesicles on his body for the past 3 days. Despite medical evaluation, a clear diagnosis was not immediately determined. Upon admission, the leukocyte count was recorded as 20.96 × 10^9^/L on the first day, leading to the initiation of broad-spectrum antibiotic treatment. Despite the general interpretation that a positive IgG and a negative IgM indicate a previous infection, the patient’s extraordinarily elevated IgG levels, coupled with a markedly increased CMV DNA quantification, prompted us to suspect a reactivation of the CMV virus. In light of these findings, we opted for the intravenous administration of ganciclovir as part of the treatment strategy. Unfortunately,,the patient succumbed to rapidly worsening symptoms and passed away. Within one week of the patient’s demise, chickenpox gradually developed in the medical staff who had been in contact with him. In such instances, we speculate that the patient’s diagnosis should be classified as a rare case of hemorrhagic varicella.

**Conclusion:**

Swift identification and timely administration of suitable treatment for adult HV are imperative to enhance prognosis.

## Background

Hemorrhagic varicella (HV) is a rare and potentially life-threatening condition that can arise concurrently with various conditions, including immunodeficiency syndrome, hematologic malignancy, and autoimmune disease [1]. Despite its potential severity, adult hemorrhagic varicella is frequently overlooked due to its low incidence rate, with only four reported cases in the English literature [2–4]. This rarity may result in the omission of this disease from the differential diagnosis when evaluating patient symptoms, potentially leading to delayed and inaccurate diagnoses, and adverse outcomes. To address this, it is imperative to raise awareness of this condition.

Cytomegalovirus (CMV)belongs to the ubiquitous herpes family. Following primary infection, CMV establishes latency but can be reactivated by various triggers. Reactivation may occur due to a range of stimuli or conditions, including immunocompromising states, drug eruptions [5], administration of immunosuppressants, HIV infection, and septic shock.

The reactivation of CMV and the infection of Varicella Zoster Virus (VZV) is linked to a poor prognosis, leading to the development of conditions such as pneumonia, colitis, hepatitis, or encephalitis. This case report aims to elaborate on the clinical features of the reactivation of HV and CMV, thereby raising awareness among healthcare professionals.

## Case presentation

**Fig. 1:**
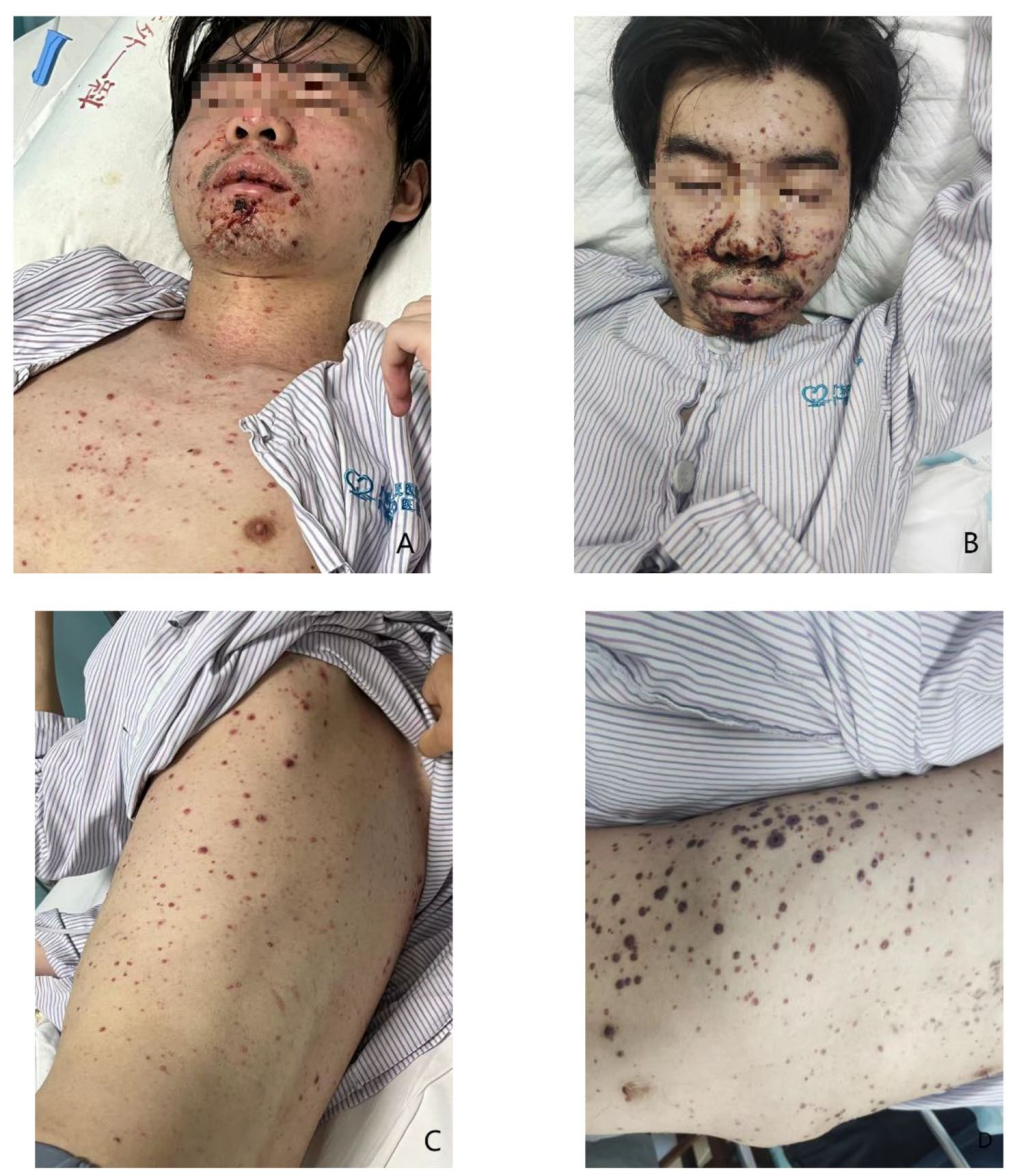
**A** and **B**: In comparison between the conditions on day 1 and day 3 of hospitalization, there was a rapid increase in lesions on the patient’s face, accompanied by a noticeable deterioration in consciousness. **C** and **D**: In comparison between day 1 and day 3 of hospitalization, the hemorrhagic vesicles on the patient’s trunk not only increased but also exhibited surrounding purpura

A 25-year-old Chinese male patient visited the emergency department (ED) with a 3 d history of countless hemorrhagic vesicles on his trunk and limbs and abdominal pain (Fig. 1A and B). Three days prior, the patient had developed abdominal pain and blood vesicles all over his body. He visited a local hospital and was given abdominal radiography showed no obstruction. After the examination, he had been treated with lactose and glycerin 1 d prior to his ED visit. However, the above symptoms did not alleviate.

The patient had a history of nephrotic syndrome and received orally administered prednisone, 50 mg per day for two months. The patient denied the history of varicella patients contaction, and never received VZV vaccination. There was no family history of severe chickenpox.

During physical examination in the ED, the patients vital signs were measured: Body temperature (36.7℃), blood pressure (140/90mmHg), heart rate (64 bpm), and respiratory rate (20 breaths/min). The patient had dry mouth with a size of approximately 3 mm$\times 3$mm ulcer, and enlarged and erythematous tonsis. Hemorrhagic vesicles covered her faces, and the remainder of his body was studded with countless vesicles and papules, including nearly confluent vesicles on his genital organ and the inguinal region. The vesicles were filled with bright red blood or clear fluid; only a few were crusted. An abdominal examination revealed tenderness and rebound pain localized to the lower right half of the abdomen. Cardiac and pulmonary examination findings were normal. No specific findings were observed in the remaining physical examination. A laboratory examination was performed once the patient was admitted our hospital. Due to changes in the condition, some indicators were rechecked on the third day (Tables 1 and 2).

**Table 1:** Laboratory examination on the 1d hospitalization

	Value w/units	Normal range
WBC	20.96 × 10^9^/L	3.5–9.5
Neutrophil ratio	0.841	0.4–0.75
RBC	5.67 × 10^12^/L	4.3–5.8
Hemoglobin	174 g/L	130–175
Platelet count	112 × 10^9^/L	125–350
Coagulation		
Prothrombin time	14.5s	11–15
INR	1.12	0.82 − 1.30
APTT	31.7s	23–44
CRP	9.2 mg/L	0–6.0
CREA	61.6umol/L	57–97
ALB	41.6 g/L	40.0–55.0
AST	161U/L	0–45
ALT	143U/L	0–50

**Table 2:** Laboratory examination on the 3d hospitalization

	Value w/units	Normal range
WBC	49.24 × 10^9^/L	3.5–9.5
Neutrophil ratio	0.909	0.4–0.75
RBC	1.34 × 10^12^/L	4.3–5.8
Hemoglobin	41 g/L	130–175
Platelet count	25 × 10^9^/L	125–350
Coagulation		
Prothrombin time	> 200.0s	11–15
INR	15.75	0.82 − 1.30
APTT	> 180.0s	23–44
D-Dimer	196.4ug/ml	0-0.5
FBI	< 0.25 g/L	2–4
NTproBNP	2430 pg/ml	0-125
hsTropI	1.13 ng/mL	0-0.12
IL6	> 4000.00pg/mL	< 7
PCT	1.38ng/mL	
Cytomegalovirus DNA quantification	1.36$\times$10^4^IU/mL	0-4$\times$10^2^
Anti CMV IgG	>1000AU/mL	
Anti CMV IgM	-	-
CRP	35.6 mg/L	0–6.0
CREA	210umol/L	57–97
ALB	17.7 g/L	40.0–55.0
AST	6969U/L	0–45
ALT	5336U/L	0–50

The patient’s head computed tomography (CT) was normal. Abdominal CT was performed due to persistent abdominal pain and it showed silty gallstones in the gallbladder. Chest CT suggested bilateral lung inflammation. At the 10th hour of hospitalization, given the significant increase in the patient’s WBC count, broad-spectrum antibiotics were administered. However, 1 d later, the patient exhibited worsening hemorrhagic ulceration in the oral mucosa compared to the initial presentation. The number of hemorrhagic varicella on the trunk increased, gradually affecting both lower limbs. A large area of ecchymosis, approximately 4 × 7 cm in size, appeared at the blood collection point on the left elbow joint flexion side. Concurrently, the patient developed hematuria, and a single episode of bright red stool was observed (Fig. 1C and D). Laboratory tests revealed disseminated intravascular coagulation, sepsis, hemorrhagic shock, and acute liver failure (Table 2). Unfortunately, high-throughput gene testing targeting pathogenic microorganisms could not be completed due to hemolysis in the patient’s blood sample.

Due to the potential for multiple organ failure, the patient was admitted to the Department of Infectious Disease Intensive Care Unit (ICU) on the 3d of hospitalization and received intravenous treatment with broad-spectrum antibiotics and ganciclovir at 250 mg per day. As his neurological and hepatic conditions progressively deteriorated, he required endotracheal intubation on the 3rd day of hospitalization, along with bedside continuous renal replacement therapy (CRRT). However, acyclovir combined with gamma globulin (recommended for severe chickenpox) was not administered. The assay was not performed due to the lack of availability of the test. Additionally, the patient’s immune system might have been weakened after long-term oral steroid use, rendering him more susceptible to infection.

During the ICU admission, the patient’s coagulation worsened, and infection indicator levels increased. The patient’s condition rapidly deteriorated. On the 4th day of hospitalization, the patient experienced cardiopulmonary arrest due to DIC and succumbed to the illness. On the 11th day after contact with the patient, some ICU physicians and the ED nurses who had been in contact with the patient exhibited symptoms such as vesicles, papules throughout the body, and high fever. All of they had not been vaccinated against the disease The diagnosis of chickenpox of them was made by infectious disease specialists or dermatologists based on the typical presentation of the rash. All of them were subsequently diagnosed with chickenpox. None of them reported any contact history with other varicella patients. Following the patient’s demise, we initiated an extensive discussion about the case across the entire hospital. Based on the characteristics of the patient’s symptoms, it can be clinically confirmed that the patient died from hemorrhagic varicella combined with cytomegalovirus infection.

## Discussion and conclusions

HV, a rare condition linked to uncontrolled VZV infection, is characterized by hemorrhagic vesicles surrounded by purpura and subconjunctival hemorrhage [6]. Older age and a compromised immune system are the most significant risk factors associated with the severity of varicella disease [7]. HV is less commonly known to occur in adults, and to date, only a few cases in adults have been reported. For instance, Benchat et al. documented a case of varicella in adult, however, the patient’s ecchymosis in skin and visceral hemorrhage were not typical [8].

CMV belongs to herpes family. After primary infection, latency of CMV is established, but it may be reactivated with some triggers, including immunocompromising condition, drug eruption, immunosuppressant administration, HIV infection and septic shock [9]. Additionally, reactivation of CMV with VZV is associated with poor prognosis. Only Akira et al. described a case of simultaneous reactivation of cytomegalovirus in an adult patient with varicella, however, there was a lack of description regarding the changes in liver function indicators in the patient [10].

Hemorrhagic manifestations and multiorgan failure are severe complications that can arise in the context of autoimmune diseases. During the patient’s hospitalization, we placed particular emphasis on ruling out the possibility of an autoimmune disease, as detailed in the Table 3. The patient’s autoantibodies associated with autoimmune diseases and HIV antibodies tested negative, leading us to exclude the diagnosis of an autoimmune condition.

**Table 3:** The patient’s autoantibody profile results“+” represents positive, “-” represents negative

Name of antibody	Test results
**Anti SCL-70**	-
**Anti CENP B**	-
**Anti ARPA**	-
**Anti ANA**	-
**Anti nRNP**	-
**Anti Sm**	-
**Anti SSA**	-
**Anti SSB**	-
**Anti AnuA**	-
**Anti AHA**	-
**Anti AMA2**	-
**Anti PCNA**	-
**Anti dsDNA**	-
**Anti RO-52**	-
**Anti Jo-1**	-
**HIV-Ab**	-

Our patient presented a challenging case of chickenpox, exhibiting an unusually extensive cutaneous manifestation characterized by the rapid progression of hemorrhagic vesicles and accompanying visceral complications. Notably, a significant number of medical staff from various departments, who had been in direct contact with the patient, contracted chickenpox within a short period. Remarkably, all affected individuals denied any history of exposure to other chickenpox patients. The patient’s initial manifestation of abdominal pain led to a misdiagnosis, aligning with Wong et al.‘s findings, where they reported cases of severe varicella presenting with abdominal pain. The authors emphasized the consideration of varicella in the differential diagnosis of abdominal pain, particularly in immunocompromised patients, especially when the etiology is not readily apparent [11].

The poor prognosis of this patient can be attributed to several factors. Firstly, the patient’s long-term immunocompromised state resulted from the oral administration of prednisolone, specifically 50 mg per day, aimed at managing nephrotic syndrome for a duration of at least two months. And the use of prednisone can be a factor in the reactivation of a CMV reactivation and the hemorrhagic transformation of the vesicular lesion. Secondly, the concomitant CMV reactivation exacerbated the situation, leading to severe hepatitis, deterioration in coagulation function, and a decline in platelet count [12]. Thirdly, the patient’s serum analysis revealed markedly elevated levels of PCT and IL-6, which are suggestive of a sepsis condition potentially triggered by the reactivation of CMV and concurrent infection with VZV. This septicemia could be identified as the primary cause of the patient’s demise. It is pertinent to note that the notable escalation in AST and ALT levels, with AST levels exceeding those of ALT, could be indicative of heart failure. This is corroborated by the marked elevation of troponin and BNP, which are reliable indicators of cardiac injury and heart failure, respectively. This suggests that the elevated AST and ALT levels may not be exclusively a consequence of liver failure induced by CMV infection. Additionally, the observed increase in these enzymes could also be associated with muscle breakdown, further complicating the interpretation of these laboratory findings. Ganciclovir stands as the preferred treatment for CMV [13] and has demonstrated effectiveness against VZV [14]. espite the intravenous administration of 0.25 g of ganciclovir per day starting on the third day of hospitalization, the treatment’s efficacy remained suboptimal. This could be attributed to the premature initiation of antiviral drugs and a potentially insufficient duration of treatment.

The current standard therapeutic regimen for HV involves early active antiviral treatment and symptomatic support, which holds significant importance in improving prognosis, particularly for immunocompromised patients (Table 4). Acyclovir is the preferred antiviral therapy for VZV infection, with an oral bioavailability ranging from 15-30% [15]. In severe VZV infections, intravenous injection becomes necessary. Combining acyclovir with gamma globulin is considered safe and effective [16]. For patients with prolonged steroid use and severe chickenpox, the question of whether to continue steroid treatment remains controversial. Traditional textbooks consider chickenpox a relative contraindication for steroids. Some studies indicate that steroids may hinder the phagocytosis of reticuloendothelial cells, reduce antibody production, and promote VZV proliferation, leading to extensive damage to vascular endothelium and a subsequent imbalance in the coagulation and fibrinolysis systems [17]. However, other studies have suggested that physiological maintenance of steroids can avoid iatrogenic adrenal insufficiency [18].

**Table 4:** Drug use during hospitalization. Some drugs used to support treatment are not listed, such as 5% glucose sodium chloride injection, potassium chloride injection, etc

The name of the drug	dose	Use frequency	Usage	The time of starting to use the drug (on which day of hospitalization)	Usage Times
Magnesium isoglycyrrhizinate injection	150 mg	QD	Intravenous Drip	1	2
Ceftriaxone Sodium for Injection	2 g	BID	Intravenous Drip	1	4
Esomeprazole Sodium for Injection	40 mg	QD	Intravenous Drip	2	1
Glutathione for injection	1.8 g	QD	Intravenous Drip	2	1
Meropenem for injection	1 g	Q8h	Intravenous Drip	3	6
Vancomycin hydrochloride for injection	1 g	Q12h	Intravenous Drip	3	1
Ganciclovir for injection	0.25 g	Q12h	Intravenous Drip	3	1
Ulinastatin for injection	300,000Units	Q8h	Intravenous Injection	4	1

In conclusion, HV is a severe form of varicella that often manifests in immunocompromised populations, posing a potential fatality risk if treatment is delayed. Patients may present with abdominal pain symptoms, prompting visits to the ED. Emergency medicine physicians should be mindful that unexplained abdominal pain may be attributed to chickenpox. Reactivation of VZV with CMV can intensify liver failure, heightening the risk of mortality. Early initiation of antiviral treatment and immunoglobulin therapy has proven efficacy. Additionally, if deemed necessary, medical staff should consider vaccination against chickenpox.

## Data Availability

Data is provided within the manuscript or supplementary information files.

## Abbreviations

HV: Hemorrhagic varicella
CMV: Cytomegalovirus
VZV: Varicella Zoster Virus
ED: Emergency Department
WBC: White blood cell
RBC: Red blood cell
INR: International standard ratio
APTT: Activated partial thromboplastin time
FBI: Fibrinogen
PCT: Procalcitonin
CRP: C-reaction protein
CREA: Creatinine
ALB: Albumin
ALT: Alanine aminotransferase
AST: Aspartate amino transferase
CT: Computed tomography
ICU: Intensive Care Unit
CRRT: Continuous renal replacement therapy
